# Longevity Attainable

**Published:** 1861-02

**Authors:** 


### HALL'S JOURNAL OF HEALTH.

**Our Legitimate Scope is almost boundless: for whatever begets pleasurable and harmless feelings, promotes Health; and whatever induces disagreeable sensations, engenders Disease.**

WE AIM TO SHOW HOW DISEASE MAY BE AVOIDED, AND THAT IT IS BEST, WHEN SICKNESS COMES, TO TAKE NO MEDICINE WITHOUT CONSULTING A PHYSICIAN.

Vol. VIII.] FEBRUARY, 1861. [No. 2.

### LONGEVITY ATTAINABLE.

MAN is like a well-adjusted and well-made machine, which, if worked steadily, will last a long time, but if moved by fits and starts, and badly cared for, will soon be jolted to pieces. Thus it is that equanimity of mind and steady bodily habits are each promotive of long life; and, when combined, will not only enable the possessor to live within sight of his century, but do it in enjoyable health of body, and a pleasurable and hilarious mental activity. Surely such an old age is worth laboring for; and that it is attained by whole classes of persons who make moderation their life-long habit, is susceptible of undeniable proof.

During the year eighteen hundred and sixty, twenty-four British peers died at an average age of three-score years and ten. During the same time, the ages of twenty-four members of the Society of Friends, whose deaths were recorded in the *Friend*, published in Philadelphia, averaged eighty-eight and a half years. Fifty lives in the same year averaged eighty-five years. The last census shows that five times as many negro slaves, in the South, reach a hundred years, as do whites. Now, through Quaker, and peer, and slave, one trait of mind is overshadowing—it is complete deliverance from the fear of physical want. The peer knows that he is provided for. It is a part of the slave's nature, from life-long habit, to lean on the master for support; hence, wearing anxiety for to-morrow's bread for himself and his children, is the very least of all his troubles. While the Friend, by a habitual reliance on Providence, and his own consistent efforts for support, grows into the faith firm as the rock of ages: "The Lord is my Shepherd, I shall not want." Beautiful truth, priceless beyond the diadems and crowns of kings!

The peer drinks wine, dines heartily and late, retiring not until the small hours of the morning come; he leaves his chamber at ten or noon. But whatever of ill is found in habits like these is more than counteracted by the abiding and calming conviction that he is amply provided for to the end of his days. He is not dunned to death for money; he is not irritated by the cheateries of men, and their equivocations and procrastinations of payment of rents; nor, indeed, is his heart saddened by the knowledge of the grinding economies and painful strainings, which even worthy tenants have but too often to encounter, to make up their quarterly payments; his agents shoulder all these disagreeables. In addition, he will not allow himself to be driven to exposures to rain and storm, or snow, or melting sun. He allows no emergency to make him almost work his life out of him, to gain a certain end half an hour sooner, as we impulsive and impatient Americans do. In fact, an English peer considers hurry a disgrace, as altogether unbecoming his station; he prides himself on his equanimity.

The slave's highest ambition is to have a plenty to eat, and a warm place to sleep in; and knowing that it is his master's business to provide these things, he has no consuming cares, and he, too, can boast of an equanimity to which his master is an absolute and a life-long stranger. The slave's equanimity is passive, arising from the want of ambition; the peer's from pride; the Quaker's from a holy, calming, and abiding trust in God, and a spotless integrity. All three—peer, Quaker, slave—have exemption from worldly care—from eating, wearing harassments. Not that Friends have nothing to annoy, but that they have a principle within which subdues annoyances, or a faith that molds them into valuable lessons. An English peer has a dignity which keeps him in the higher regions where storms do not come. The slave, like the child, lives in a happy unconsciousness of anxieties, and, like the child also, has such a buoyancy of nature, that a tear has hardly time to clear itself before the smile comes to chase it away. A child will play; a slave will sing. Equanimity of mind, then, is the great catholicon of humanity. Let all who would have length of days, whatever may be their station in life, strive for an equable frame of mind, for an implicit reliance that temperance, integrity, industry, resignation, and godliness, not only "have the promise of the life that now is, but of that which is to come."

